# Effects of collagen peptide supplementation on bone turnover, cytokine, and inflammatory markers in female distance runners: a randomized pilot study

**DOI:** 10.3389/fnut.2026.1825906

**Published:** 2026-05-14

**Authors:** Nicholas M. Kuhlman, Andrew R. Jagim, Christopher N. Blesso, Michael J. Puglisi, Ock K. Chun, Margaret T. Jones, Jennifer B. Fields

**Affiliations:** 1 Exercise and Sport Science Department, Fitchburg State University, Fitchburg, MA, United States; 2 Department of Nutritional Sciences, University of Connecticut, Storrs, CT, United States; 3 Sports Medicine Department, Mayo Clinic Health System, La Crosse, WI, United States; 4 Patriot Performance Laboratory, Frank Pettrone Center for Sports Performance, George Mason University, Fairfax, VA, United States; 5 Sport, Recreation, and Tourism Management, George Mason University, Fairfax, VA, United States

**Keywords:** bone turnover marker, collagen peptide, cytokine, distance running, inflammation

## Abstract

**Introduction:**

Female distance runners are at elevated risk for impaired bone remodeling due to high mechanical loading, potential low energy availability, and sustained inflammatory stress. Collagen peptide (CP) supplementation has been proposed as a nutritional strategy to support type I collagen synthesis and modulate osteoimmune signaling; however, evidence in premenopausal endurance athletes is limited. This pilot randomized, double-blind, placebo-controlled trial examined the effects of four weeks of high-dose CP supplementation on markers of bone metabolism and inflammatory activity in endurance-trained premenopausal women.

**Methods:**

Twenty-two participants (18–35 years; ≥35 miles/week [>56km/week]) were randomized to CP (INT; 20 g/day) or isocaloric maltodextrin (CON). Pre- and post-intervention assessments, conducted in the early follicular phase, included serum procollagen type I N-terminal propeptide (P1NP), plasma C-terminal telopeptide of type I collagen (CTX-1), serum soluble receptor activator of nuclear factor-κB ligand (sRANKL), osteoprotegerin (OPG), the sRANKL/OPG ratio, and interleukin-6 (IL-6). Repeated-measures ANCOVAs were performed, adjusting for accumulated running distance and vitamin D status.

**Results:**

A significant group × time interaction was observed for P1NP (*p* = 0.04), with increases in INT and no change in CON. No significant interaction was observed for CTX-1 (*p* = 0.13). Significant interactions were also observed for sRANKL (*p* = 0.046) and IL-6 (*p* = 0.03). No significant interaction effects were detected for sRANKL, OPG, or the sRANKL/OPG ratio.

**Discussion:**

Short-term CP supplementation increased a marker of bone formation, altered osteoclast-related signaling, and reduced IL-6 in endurance-trained premenopausal women. These findings support the potential for CP-mediated modulation of bone turnover and inflammatory activity and warrant further investigation in larger, adequately powered trials incorporating structural bone outcomes.

## 1 Introduction

Distance running is a high-volume, weight-bearing sport that subjects the skeletal system to repetitive mechanical loading (1). While such loading is necessary to stimulate bone adaptation, excessive frequency, inadequate recovery, and suboptimal fueling can disrupt the balance between microdamage formation and repair, increasing susceptibility to bone stress injuries (BSIs) (2). BSIs are common in competitive runners, with 30–60% reporting a prior history (3, 4), and female athletes experience approximately twice the risk observed in males (2). Up to 20% of female distance runners may sustain a BSI annually (5). This heightened vulnerability reflects the convergence of high mechanical strain, hormonal variability, and potential periods of low energy availability (LEA), all of which can impair bone remodeling and compromise skeletal integrity (6–8).

Bone remodeling is governed by the coordinated activity of osteoclast-mediated resorption and osteoblast-driven formation. Repetitive loading initiates targeted remodeling to repair microdamage; however, when damage accumulation outpaces repair, bone strength declines (1). This process is further influenced by inflammatory signaling. Cytokines such as interleukin-6 (IL-6) can promote osteoclastogenesis, in part by altering the balance between receptor activator of nuclear factor-κB ligand (RANKL) and osteoprotegerin (OPG), a key regulatory axis controlling bone resorption (9). Endurance training, particularly under conditions of LEA, may contribute to a chronically elevated inflammatory tone that shifts this balance toward catabolism (10–12). Thus, interventions capable of simultaneously supporting bone formation and modulating pro-resorptive inflammatory signaling are of particular relevance in female endurance athletes.

Nutritional strategies to protect skeletal health typically emphasize adequate protein, calcium, and vitamin D intake (13). Although these nutrients support mineralization and overall bone metabolism, they may not directly target collagen synthesis within the organic bone matrix or upstream immunoskeletal pathways. Collagen peptides (CPs), derived from enzymatically hydrolyzed type I collagen, have gained attention as a potential adjunct strategy. CPs are rich in glycine, proline, and hydroxyproline and can be absorbed as bioactive di- and tripeptides that circulate systemically and may stimulate osteoblast activity and extracellular matrix synthesis (14–17). Clinical studies in postmenopausal women suggest that CP supplementation can favorably influence bone turnover markers and, in some cases, bone mineral density (18), while evidence for modulation of inflammatory mediators remains limited and inconsistent, with several studies reporting no significant effects on markers such as IL-6 and RANKL/OPG signaling (19–21), primarily in male or mixed-sex cohorts and non-endurance-trained populations.

Despite these promising findings, research in premenopausal female endurance athletes remains limited. Most investigations have focused on postmenopausal populations or male cohorts, whose physiological and mechanical contexts differ from those of young, endurance-trained women. Female distance runners are exposed to repeated high-impact loading (1), cyclical fluctuations in sex hormones (22), and an elevated risk of low energy availability (23), each of which can independently and interactively influence bone turnover and inflammatory signaling. These factors create a distinct physiological environment in which osteoblast and osteoclast activity, as well as cytokine dynamics, may respond differently to nutritional interventions compared to other populations. However, few short-term randomized trials have examined dynamic bone turnover markers (BTMs) such as procollagen type I N-terminal propeptide (P1NP) and C-terminal telopeptide of type I collagen (CTX-1), alongside inflammatory mediators in this group. As a result, it remains unclear whether CP supplementation can modulate the osteoimmune interface under conditions of sustained mechanical and metabolic stress characteristic of endurance training. Thus, the purpose of this randomized, double-blind, placebo-controlled pilot trial was to determine the effects of 4 weeks of high-dose CP supplementation on markers of bone metabolism (P1NP, CTX-1) and inflammatory signaling (sRANKL, OPG, the sRANKL/OPG ratio, and IL-6) in endurance-trained premenopausal women.

## 2 Materials and methods

### 2.1 Experimental design

A prospective, randomized, double-blind, placebo-controlled, parallel-group pilot trial with a 1:1 allocation ratio was designed to assess the effects of CP supplementation on bone turnover, cytokine, and inflammatory markers in premenopausal female endurance runners. The study is reported in accordance with CONSORT guidelines for randomized controlled trials. No important changes to the study methods were made after trial commencement. The study was conducted at the University of Connecticut in Storrs, CT, USA, with all laboratory assessments performed in a controlled research setting. Participant recruitment and data collection were conducted between November 2024 and April 2025.

### 2.2 Participants

Inclusion criteria were: female participants aged 18–35 years, naturally menstruating (defined as self-reported menstrual cycles of 21–35 days over the previous 3 months, without biochemical confirmation of ovulation), and a minimum running volume of ≥35 miles/week (≥56 km/week) (24–26). Naturally menstruating status was required because (1) menstrual cycle phase influences BTM levels, particularly during the luteal phase (25, 27), and (2) the study’s 28-day intervention was designed to align with the typical menstrual cycle duration (24, 28). The minimum running volume criterion aligns with established thresholds used in prior studies of distance runners (29–31) and was selected to ensure inclusion of participants exposed to sufficient mechanical loading, while also maintaining recruitment feasibility within the target population. These criteria ensured that the study population was representative of premenopausal female distance runners at risk for maladaptive bone responses, enhancing the relevance and applicability of the findings.

Exclusion criteria included amenorrhea, oligomenorrhea, a known history of reproductive disorders, pregnancy or the intention to become pregnant, and an age under 18 or over 35 years (24). Participants with a history of metabolic bone disease, hormonal disorders, or bone fractures in the last 12 months, those who consumed more than two units of alcohol per day, or smokers were also excluded (32). Additionally, individuals who had taken medications that affect bone metabolism within the last year, such as oral anti-acne medications, oral anti-psoriasis medications, medroxyprogesterone acetate, norethindrone, and gonadotropin-releasing hormone analogs, were excluded (25, 33). The exclusion criteria also included the use of long-acting hormones, anabolic steroids/androgens, and aromatase inhibitors within the past 3 years (25, 32). Further, participants who had used bisphosphonates, methotrexate, calcineurin inhibitors, tumor necrosis factor inhibitors (e.g., etanercept), selective estrogen receptor modulators (e.g., raloxifene, tamoxifen), calcitonin, or teriparatide within the past 5 years were excluded (25, 32). Finally, participants who were currently consuming collagen supplements were required to discontinue use for a four-week washout period before participating.

### 2.3 Procedures

#### 2.3.1 Participant recruitment and randomization

**Figure 1:**
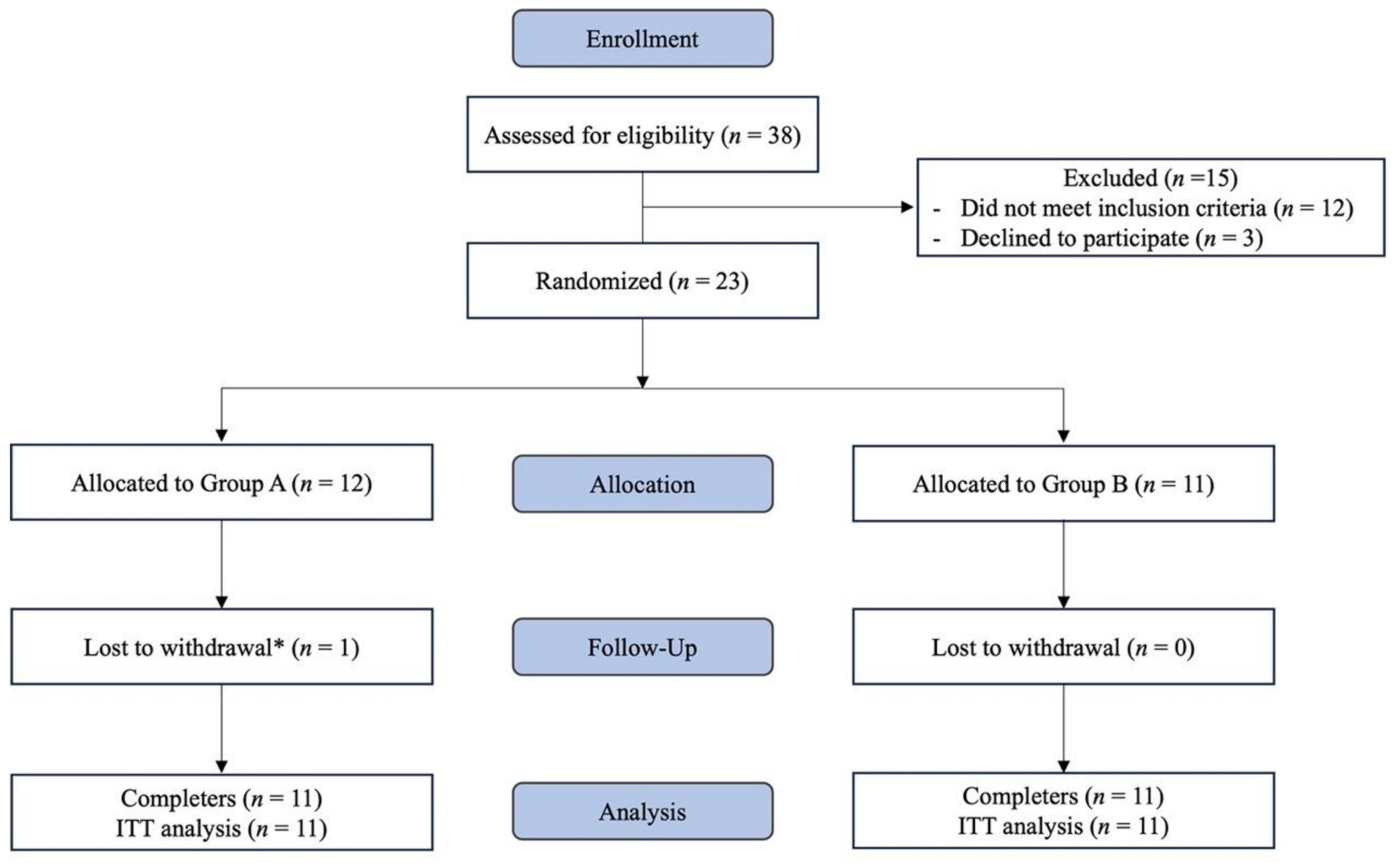
Diagram of subject recruitment, enrollment, allocation, follow-up, and intended analysis. ITT: intention-to-treat analysis.

A total of 38 participants were recruited and screened based on inclusion and exclusion criteria. Of these, 23 met eligibility requirements and were randomized. One participant withdrew on Day 3 prior to initiation of the intervention, resulting in a final sample of 22 participants who completed the study. Eligible participants were randomly assigned to one of two groups: (1) intervention (INT) or (2) control (CON) group (Figure 1). Randomization was conducted using the NIH Clinical Trial Randomization Tool, which generated a computer-based random allocation sequence using block randomization to assign participants to each group in a 1:1 ratio (34) (Figure 1). The random allocation sequence was generated by the primary investigator, who also enrolled participants. Group allocation was concealed from both participants and investigators at the time of enrollment, with supplement assignments pre-prepared and coded to maintain blinding.

#### 2.3.2 Supplementation protocol

Participants in the INT group received 20 g of CP (Vital Proteins, Chicago, Illinois, USA) daily, while those in the CON group received 20 g of isocaloric, carbohydrate-matched maltodextrin (NOW Foods, Bloomingdale, Illinois, USA). Detailed amino acid profiling specific to the collagen peptide product was not available from the manufacturer; however, the product is composed predominantly of glycine, proline, and hydroxyproline, consistent with typical collagen-derived peptide formulations. Participants were instructed to consume their assigned supplement within 1 h of waking to standardize intake timing, and to document the timing of ingestion daily. Both supplements were indistinguishable in color, appearance, smell, and taste, and were provided in identical packaging to maintain the integrity of the double-blind design. Participants and investigators involved in data collection and analysis were blinded to group allocation throughout the study. Compliance was monitored through a shared Microsoft Office365 document, where participants recorded the timing and date of each supplement ingestion.

#### 2.3.3 Dietary and exercise monitoring

**Figure 2:**
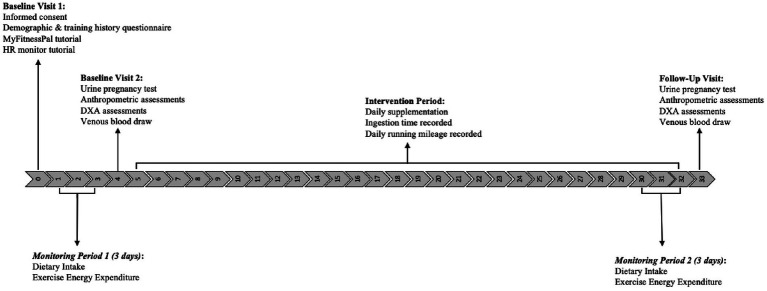
Study timeline. HR: heart rate; DXA: dual-energy x-ray absorptiometry.

Energy intake (EI) and exercise energy expenditure (EEE) were assessed during two 3-day periods: (1) the 3 days preceding the intervention and (2) the final 3 days before the last laboratory visit (Figure 2). These periods were selected to capture habitual EI and training patterns immediately preceding each blood collection, recognizing that inclusion of a weekend day was not always feasible due to the timing of laboratory visits within the menstrual cycle. Throughout the 28-day intervention, participants logged their daily running mileage in a shared Microsoft Office365 document. Mileage was verified using data from participants’ personal tracking devices or applications (e.g., Strava, Garmin, Apple Watch), which they were instructed to use consistently throughout the study. Reported mileage was reviewed for plausibility and cross-referenced with participants’ tracking data when available to ensure consistency and accuracy.

Participants logged their EI and EEE during both designated 3-day monitoring periods. Each day, they recorded dietary intake, including total calories (kcal), carbohydrates (g), protein (g), and fat (g), using the MyFitnessPal™ app (35, 36). EEE (kcal) during distance running training sessions or competitions was tracked using a Polar H10 heart rate monitor (37) to facilitate the calculation of energy availability (EA). Participants entered these data into the shared Microsoft Office 365 document. While the Polar H10 provides reliable heart rate data, its use in estimating EEE is based on proprietary algorithms and lacks direct validation for precise quantification of energy expenditure, particularly in free-living exercise settings, which is acknowledged as a limitation. EA was estimated as:


$$\mathrm{EA}=\frac{\begin{array}{c} \text{Energy intake}\;(\text{kcal}\cdot {\mathrm{day}}^{-1})\\ -\text{Exercise Energy Expenditure}\;(\text{kcal}\cdot {\mathrm{day}}^{-1}) \end{array}}{\mathrm{Fat}-\text{free mass}\;\;(\mathrm{kg})}$$

EI was derived from dietary logs, EEE was estimated from heart rate monitoring, and fat-free mass (FFM) was obtained from dual-energy X-ray absorptiometry (DXA). EA values were averaged across each 3-day monitoring period for descriptive analysis.

#### 2.3.4 Laboratory visits

Participants attended three laboratory visits throughout the study (Figure 2). The first visit (Day 0) included obtaining informed consent, completing a demographic and training history questionnaire, and providing participants with detailed instructions for tracking their dietary intake and exercise energy expenditure during the two designated 3-day monitoring periods. Heart rate monitors were issued during this visit to accurately calculate EEE during these periods.

The second visit (Day 4) consisted of a comprehensive set of assessments. Participants first underwent a urine pregnancy test, followed by anthropometric measurements, and DXA scans to evaluate bone mineral density (BMD) and body composition. A venous blood draw was then conducted to analyze biomarkers, focusing on bone turnover, cytokine, and inflammatory markers. Participants were instructed to refrain from exercise for 24 h prior to the visit to minimize the influence of acute exercise on biomarker concentrations. At the conclusion of the visit, participants received their 28-day supply of supplements.

The third and final visit (Day 34) included anthropometric assessments and a venous blood draw to analyze the same bone turnover, cytokine, and inflammatory biomarkers. Participants were again required to refrain from exercise for 24 h prior to sample collection. Both Visit 2 and Visit 3 took place during the first 7 days of each participant’s menstrual cycle, aligning with the early to mid-follicular phase (25, 27). Consistent timing of blood collections was crucial due to cyclic variations in BTMs observed during the menstrual cycle, which are likely associated with cyclic fluctuations in estradiol and progesterone (22, 38–40).

#### 2.3.5 Anthropometric assessments

Body mass and height were assessed using a self-calibrating physician’s scale (Detecto, Webb City, MO, USA) and stadiometer (Detecto, Webb City, MO, USA) to the nearest 0.1 kg and 0.5 cm, respectively.

#### 2.3.6 DXA

Participants underwent BMD and body composition assessments using DXA (Hologic, Horizon A, Hologic Inc., Waltham, MA, USA). The DXA machine was calibrated daily before use with the manufacturer-provided calibration block. Participants were asked to remove any metal or metal-containing clothing before the measurement. Scans were conducted on the left hip, lumbar spine, and whole body, with participants positioned supine on the DXA scanning bed according to the manufacturer’s instructions. They were required to remain still during each assessment. Three scans were performed, including the hip (~1 min), spine (~1 min), and whole body (~3 min), for a total duration of approximately 5 min. BMD (whole body, total hip, spine), total fat mass (FM), FFM, and body fat percentage (BF%) were recorded for each participant to characterize body densitometry and composition. These data were used descriptively to characterize the study population.

#### 2.3.7 Venous blood draw and biomarker measurement

Participants arrived between 7:30 and 10:00 a.m. (41), following a 12-h fast, which included abstaining from alcohol and caffeine (18). An overnight fast has been shown to markedly reduce the circadian variation of CTX-1 in premenopausal women, from 40 to 16% (42, 43). Participants were also required to be in a rested state, refraining from exercise the day prior to testing (41). While this standardized sampling window was implemented to minimize biological variability, some residual diurnal variation in CTX-1 may persist and is acknowledged as a limitation (42). Blood samples were collected from an antecubital forearm vein into ethylenediaminetetraacetic acid (EDTA) or serum tubes, depending on the biomarker of interest. Plasma samples obtained from EDTA tubes were used for the analysis of CTX-1, while serum samples were used for the analysis of P1NP, sRANKL, OPG, IL-6, and 25(OH)D_3_. Following collection, samples were processed and stored according to standard laboratory procedures prior to analysis.

All biomarkers were quantified via enzyme-linked immunosorbent assay (ELISA) using commercially available kits, following manufacturer instructions. P1NP (Antibodies.com, A78632), CTX-1 (FineTest^®^, EH0996), sRANKL (Antibodies.com, A302604), OPG (Antibodies.com, A78324), IL-6, (Antibodies.com, A78324), and 25(OH)D_3_ (Invitrogen, EEL152) were measured using assay-specific kits. All assays were performed in duplicate, and concentrations were determined using standard curves generated via four-parameter logistic (4-PL) regression.

#### 2.3.8 Daily supplementation

Participants were randomly assigned to either INT or CON in a double-blind manner and received 28 individual bags containing either 20 g of CPs or 20 g of maltodextrin (placebo). The 28-day supply was provided at Laboratory Visit 2 (Day 4). Supplements were provided in identical, unlabeled packets, and no discernible differences in flavor, texture, or appearance existed between conditions. Participants were not informed of group allocation. Participants were instructed to consume each daily dose with 500 mL (~2 cups) of water (44, 45) and to record the exact time of ingestion in the Microsoft Office365 shared document. This secure, real-time monitoring system enabled the research team to track adherence to the supplementation protocol. Participants were informed during the consent process that the supplement provided might be derived from animal sources and were given the opportunity to withdraw if this conflicted with personal dietary preferences (e.g., vegetarian or vegan). Participants were instructed to report any adverse events or unintended effects throughout the study period. While participants were instructed not to discuss study procedures with others, it is possible that some individuals (e.g., teammates) may have communicated about their supplementation, which is acknowledged as a potential limitation.

#### 2.3.9 Daily training log

Participants recorded their daily running mileage in the Microsoft Office365 shared document for the entire 28-day intervention period. This allowed both participants and the research team to monitor training volume during the intervention.

#### 2.3.10 Statistical analysis

The primary outcomes were serum P1NP and plasma CTX-1. Secondary outcomes included serum sRANKL, OPG, the sRANKL/OPG ratio, and IL-6.

Statistical analyses were conducted using RStudio (version 2025.05.0 + 496) (*p* < 0.05). Student’s *t*-tests were used to compare baseline values between study groups (INT and CON) for all demographic variables, anthropometric variables (e.g., body mass, height, body composition, bone mineral density), EI, EEE, and EA. If the assumption of equal variances was violated, as determined by Levene’s test, Welch’s *t*-test were applied instead.

For primary outcomes, separate repeated-measures ANCOVAs were conducted to compare pre- to post-intervention changes in P1NP and CTX-1 between INT and CON, controlling for accumulated running volume (total mileage) during the 28-day intervention and baseline 25(OH)D₃ status. Exploratory analyses compared change scores (ΔP1NP, ΔCTX-1) between groups using independent-samples *t-*tests and one-way ANCOVAs, with the latter adjusted for the same covariates.

For secondary outcomes, separate repeated-measures ANCOVAs were conducted to compare pre- to post-intervention changes in sRANKL, OPG, the sRANKL/OPG ratio, and IL-6 between INT and CON, controlling for the same covariates. Exploratory analyses compared change scores (ΔsRANKL, ΔOPG, ΔsRANKL/OPG ratio, ΔIL-6) between groups using independent-samples *t*-tests and one-way ANCOVAs, with the latter adjusted for the same covariates.

No additional subgroup analyses were performed. No changes to primary or secondary outcomes were made after trial commencement. Effect sizes were calculated for all relevant analyses to estimate the magnitude of observed effects. Partial eta squared (*η*^2^*_p_*) values were interpreted as small (0.01), medium (0.06), or large (0.14) (45). Cohen’s *d* values were interpreted as small (0.20), medium (0.50), and large (0.80) (46). In addition, 95% confidence intervals (CIs) were reported for all parameter estimates, effect sizes, and mean differences. As this was an exploratory pilot study, no formal *a priori* sample size calculation was performed. The sample size was determined based on feasibility and recruitment capacity within the study timeframe. No interim analyses or stopping guidelines were planned or conducted.

Covariates were selected based on their physiological relevance to the primary and secondary outcomes. Baseline 25(OH)D_3_ was included to account for interindividual variability in vitamin D status, which influences bone remodeling through its role in calcium absorption and availability, osteoblast differentiation (46), and regulation of osteoclastogenesis (47). Vitamin D signaling also modulates immune cell activity and cytokine production (48). Accumulated running volume during the intervention was included to account for differences in cumulative mechanical loading, which is a potent stimulus for bone adaptation (49). Repetitive loading associated with endurance running activates osteocyte-mediate mechanotransduction pathways, influencing both bone formation and resorption, and can also elicit transient changes in inflammatory mediators in response to tissue stress and recovery demands (1). The assumptions of ANCOVA were evaluated prior to analysis. Homogeneity of regression slopes were assessed by testing covariate × group interactions, and no significant interactions were observed. Both visual inspection and statistical evaluation of residuals indicated no deviations from normality or homoscedasticity.

## 3 Results

No participants were lost to follow-up or excluded after randomization; therefore all 22 randomized participants were included in the final analyses.

### 3.1 Demographic characteristics, body composition, bone mineral density, and accumulated running distance

A summary of participant demographics, body composition, and BMD is presented in Table 1. No differences were observed between the CON and INT groups across age, anthropometric characteristics, or BMD measures (all *p*-values > 0.05). Additionally, no difference was observed in accumulated distance across the intervention between groups (CON: 200 ± 87 km, range: 100–385 km; INT: 179 ± 58 km, range: 101–263 km; *p* = 0.51, *d* = 0.28, 95% CI: [−0.61, 1.17]). Similarly, group differences across Weeks 1–4 were not significant (all *p*-values > 0.05), though moderate effect sizes were observed (Week 1: 0.50 [−0.40, 1.41]; Week 2: 0.77 [−0.16, 1.69]; Week 3: 0.55 [−0.36, 1.45]; Week 4: 0.55 [−0.36, 1.45]).

**Table 1:** Demographic characteristics and baseline body composition and bone mineral density.

Characteristic	Variable	All (*n* = 22)	CON (*n* = 11)	INT (*n* = 11)	Between group *p*-value
Age (y)		23.2 ± 4.2	24 ± 3.6	22.4 ± 4.8	0.38
Body Composition	Height (m)	1.68 ± 0.1	1.7 ± 0.1	1.6 ± 0.1	0.23
	Mass (kg)	62.5 ± 6.6	63.7 ± 7.8	61.3 ± 5.0	0.42
	BMI (kg/m^2^)	22.1 ± 2.4	22.1 ± 2.9	22.1 ± 1.8	0.96
	FM (kg)	17.1 ± 3.8	17.7 ± 4.2	16.6 ± 3.6	0.58
	FFM (kg)	46.0 ± 5.0	46.9 ± 5.4	45.1 ± 4.6	0.43
	FFMI (kg/m^2^)	16.3 ± 1.7	16.3 ± 1.9	16.3 ± 1.5	0.99
	Body fat (%)	27.1 ± 4.6	27.2 ± 4.1	26.9 ± 5.3	0.92
BMD (*z*-score)	Whole body	0.8 ± 0.9	0.9 ± 0.8	0.7 ± 0.1	0.59
	Hip	0.8 ± 0.9	0.8 ± 0.9	0.7 ± 0.8	0.91
	Femoral neck	0.5 ± 0.9	0.6 ± 0.9	0.4 ± 0.9	0.62
	Spine (L1–L4)	−0.2 ± 0.9	−0.1 ± 0.6	−0.3 ± 1.3	0.80
	L1	0.0 ± 0.9	0.1 ± 0.6	−0.2 ± 1.2	0.56
	L2	0.2 ± 0.9	0.3 ± 0.6	0.0 ± 1.2	0.57
	L3	−0.2 ± 0.9	0.0 ± 0.8	−0.3 ± 1.2	0.56
	L4	−0.6 ± 0.9	−0.7 ± 0.7	−0.4 ± 1.1	0.51

Values presented as mean ± SD. INT: intervention; CON: control; BMD: bone mineral density; BMI: body mass index; FM: fat mass; FFM: fat-free mass; FFMI: fat-free mass index.

### 3.2 Baseline dietary intake, exercise energy expenditure, and energy availability

Baseline dietary intake, exercise energy expenditure, and energy availability over the initial 3-day monitoring period (days 1–3 of the study) are presented in Table 2. No differences were observed between INT and CON groups for EI, macronutrient intake (both absolute and relative to body mass), EEE, EA, or 25(OH)D_3_ status (all *p*-values > 0.05).

**Table 2:** Baseline dietary intake, exercise energy expenditure, energy availability, and Vitamin D_3_ status across first 3-day monitoring period.

Characteristic	Variable	All (*n* = 22)	CON (*n* = 11)	INT (*n* = 11)	Between group *p*-value
Energy intake	Energy (kcal/d)	2,144 ± 599	2,080 ± 575	2,208 ± 643	0.63
	Relative energy (kcal/kg/d)	34.7 ± 10.3	33.3 ± 10.1	37.1 ± 10.5	0.53
	Carbohydrate (g/d)	252 ± 72	255 ± 79	249 ± 69	0.84
	Relative carbohydrate (g/kg/d)	4.07 ± 1.2	4.09 ± 1.4	4.0 ± 1.0	0.92
	Protein (g/d)	98.7 ± 36.9	94.3 ± 37.6	103.1 ± 37.4	0.59
	Relative protein (g/kg/d)	1.6 ± 0.6	1.5 ± 0.5	1.7 ± 0.7	0.39
	Fat (g/d)	80.5 ± 24.8	76.7 ± 20.4	84.3 ± 28.9	0.48
	Relative fat (g/kg/d)	1.3 ± 0.4	1.2 ± 0.4	1.4 ± 0.5	0.45
Energy expenditure	EEE (kcal)	606 ± 238	660 ± 243	552 ± 233	0.30
Energy status	Energy availability (kcal/kg FFM/d)	33.5 ± 13.2 (range: 5.8–60.3)	30.6 ± 14.1 (range: 5.8–51.6)	36.5 ± 12.3 (range: 20.8–60.3)	0.31
	<30 kcal/kg FFM/d, *n* (%)	8 (36%)	5 (45%)	3 (27%)	-
	30–44 kcal/kg FFM/d, *n* (%)	11 (50%)	5 (45%)	6 (55%)	-
	>45 kcal/kg FFM/d, *n* (%)	3 (14%)	1 (9%)	2 (18%)	-
Vitamin D_3_ status	25(OH)D_3_ (ng/mL)	41.9 ± 19.9	44.6 ± 26.2	39.1 ± 11.1	0.52

Values represented as mean ± SD. INT: intervention; CON: control; EEE: exercise energy expenditure; FFM: fat-free mass; 25(OH)D_3_: 25-hydroxyvitamin D_3_.

### 3.3 Follow-up dietary intake, exercise energy expenditure, and energy availability

Follow-up dietary intake, exercise energy expenditure, and energy availability over the final 3-day monitoring period (days 30–32 of the study) are presented in Table 3. No differences were observed between INT and CON groups for EI, macronutrient intake (both absolute and relative to body mass), EEE, EA, or 25(OH)D_3_ status (all *p*-values > 0.05).

**Table 3:** Dietary intake, exercise energy expenditure, and energy availability status across the follow-up 3-day monitoring period.

Characteristic	Unit	All (*n* = 22)	CON (*n* = 11)	INT (*n* = 11)	Group comparison *p*-value
Energy intake	Energy (kcal/d)	2,112 ± 453	2,061 ± 394	2,164 ± 519	0.61
	Relative energy (kcal/kg/d)	34.0 ± 8.2	32.9 ± 7.9	35.1 ± 8.7	0.54
	Carbohydrate (g/d)	251.3 ± 77.5	256.1 ± 67.8	246.5 ± 89.3	0.78
	Relative carbohydrate (g/kg/d)	4.05 ± 1.4	4.09 ± 1.3	4.0 ± 1.5	0.86
	Protein (g/d)	102.9 ± 48.8	94.9 ± 38.7	110.9 ± 58.0	0.46
	Relative protein (g/kg/d)	1.7 ± 0.8	1.5 ± 0.5	1.8 ± 1.1	0.34
	Fat (g/d)	84.2 ± 41.1	76.2 ± 15.5	92.3 ± 56.3	0.38
	Relative fat (g/kg/d)	1.4 ± 0.7	1.2 ± 0.3	1.5 ± 0.9	0.37
Energy expenditure	EEE (kcal)	558 ± 301	522 ± 160	594 ± 402	0.59
Energy status	Energy availability (kcal/kg FFM/d)	34.0 ± 10.6 (range: 19.8–64.1)	33.1 ± 8.8 (range: 19.8–49.7)	34.9 ± 12.4 (range: 20.3–64.1)	0.69
	<30 kcal/kg FFM/d, *n* (%)	8 (36%)	4 (36%)	4 (36%)	-
	30–44 kcal/kg FFM/d, *n* (%)	12 (55%)	6 (55%)	5 (45%)	-
	>45 kcal/kg FFM/d, *n* (%)	2 (9%)	1 (9%)	2 (9%)	-
Vitamin D_3_ status	25(OH)D_3_ (ng/mL)	46.1 ± 18.8	46.6 ± 24.6	45.7 ± 11.9	0.92

Values represented as mean ± SD. INT: intervention; CON: control; EEE: exercise energy expenditure; FFM: fat-free mass; 25(OH)D_3_: 25-hydroxyvitamin D_3_.

### 3.4 Bone turnover markers

#### 3.4.1 P1NP

**Figure 3:**
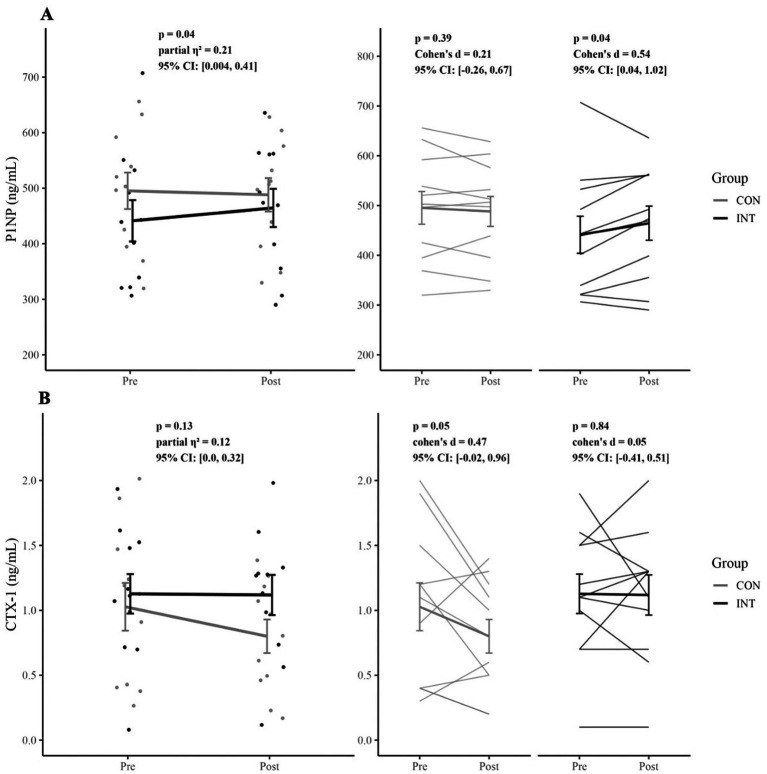
Changes in bone turnover markers from pre- to post-intervention. **(A)** Procollagen type I N-terminal propeptide (PINP) and **(B)** C-terminal telopeptide of type I collagen (CTX-1) in the control (CON) and intervention (INT) groups. Data are presented as individual participant values, with group means ± standard error. Panel A and Panel B display group-level responses, while adjacent panels illustrate individual trajectories within each group. Statistical results for time X group interactions (left panels) and within-group pre-post comparisons (right panels) are shown.

Changes in serum P1NP concentrations across the intervention period are presented in Tables 4, 5 and visualized in Figure 3. There was a significant group × time interaction (*F*(1, 18) = 4.76, *p* = 0.04, *η*^2^_p_ = 0.21, 95% CI: [0.004, 0.41]). There were no significant main effects of group (*F*(1, 18) = 0.43, *p* = 0.52, *η*^2^_p_ = 0.02, 95% CI: [0.00, 0.19], or time (*F*(1, 18) = 0.99, *p* = 0.33, *η*^2^_p_ = 0.05, 95% CI: [0.00, 0.24]. Neither accumulated running distance (*p* = 0.13) nor baseline 25(OH)D₃ status (*p* = 0.19) were significant covariates.

**Table 4:** Serum P1NP and plasma CTX-1 concentrations at pre- and post-intervention.

Variable	CON (*n* = 11)		INT (*n* = 11)		*p*-values			Effect size *η*^2^_p_ [95% CI]
	Pre	Post	Pre	Post	Group	Time	Interaction	
P1NP (ng/mL)	495 ± 109	488 ± 99	441 ± 123	464 ± 114	0.51	0.33	0.04	0.21 [0.004, 0.41]
CTX-1 (ng/mL)	1.03 ± 0.6	0.80 ± 0.4	1.13 ± 0.5	1.12 ± 0.5	0.42	0.55	0.13	0.12 [0.00, 0.32]

Values represent mean ± SD. *p*-values reflect results from separate repeated-measures ANCOVAs adjusted for accumulated running distance and baseline 25(OH)D₃ status. Effect sizes are presented as partial eta-squared (*η*^2^_p_) with 95% confidence intervals. P1NP: N-terminal propeptide of type I procollagen; CTX-1: C-terminal telopeptide of type I collagen.

**Table 5:** Change in P1NP and CTX-1 from pre- to post-intervention.

Variable	CON (*n* = 11)	INT (*n* = 11)	Between group *p*-value	Effect size Cohen’s *d* [95% CI]
Δ P1NP (ng/mL)	−7.2 ± 28.3	23.1 ± 43.7	0.07	0.82 [−0.10, 1.75]
Within-group *p*-value	0.39	0.04		
Δ CTX-1 (ng/mL)	−0.23 ± 0.4	−0.01 ± 0.4	0.24	0.52 [−0.38, 1.42]
Within-group *p*-value	0.05	0.84		

Values represent mean ± SD. Between-group comparisons reflect independent-samples *t*-tests with equal variance assumed. Within-group *p*-values were derived from post hoc comparisons in repeated-measures ANCOVA models adjusted for accumulated running distance and baseline 25(OH)D₃ status. Cohen’s *d* values and 95% confidence intervals reflect between-group effect sizes. ΔP1NP: change in N-terminal propeptide of type I procollagen; ΔCTX-1: change in C-terminal telopeptide of type I collagen.

In the CON group, mean P1NP concentrations were 495 ± 109 ng/mL (95% CI: [422, 568]) at pre-intervention and 488 ± 99 ng/mL (95% CI: [421, 555]) at post-intervention, with no significant within-group change (*p* = 0.39, *d* = 0.21, 95% CI: [−0.26, 0.67]). In contrast, the INT group showed a significant within-group increase of 5.1% in P1NP, from 441 ± 123 ng/mL (95% CI: [358, 524]) at pre-intervention to 464 ± 114 ng/mL (95% CI: [388, 541]) at post-intervention (*p* = 0.04, *d* = 0.54, 95% CI: [0.04, 1.02]).

However, there was no between-group difference in change scores (ΔP1NP) (*t*(20) = 1.93, *p* = 0.07, *d* = 0.82, 95% CI: [−0.10, 1.75]). When controlling for accumulated distance and baseline 25(OH)D_3_ status, between-group differences remained non-significant (*F*(1, 18) = 3.73, *p* = 0.07, *η*^2^_p_ = 0.17, 95% CI: [0.00, 0.37]. The CON group exhibited a mean change of −7.2 ± 28.3 ng/mL (95% CI: [−26.2, 11.8]), whereas the INT group showed a mean change of 23.1 ± 43.7 ng/mL (95% CI: [−6.2, 52.5]).

#### 3.4.2 CTX-1

Changes in plasma CTX-1 concentrations across the intervention period are presented in Tables 4, 5 and visualized in Figure 3. There were no significant main effects for group (*F*(1, 18) = 0.67, *p* = 0.42, *η*^2^_p_ = 0.04, 95% CI: [0.00, 0.22]) or time (*F*(1, 18) = 0.38, *p* = 0.55, *η*^2^_p_ = 0.02, 95% CI: [0.00, 0.18], and no significant group × time interaction (*F*(1, 18) = 2.47, *p* = 0.13, *η*^2^_p_ = 0.12, 95% CI: [0.00, 0.32]. Neither accumulated distance (*p* = 0.30) nor baseline 25(OH)D₃ status (*p* = 0.27) were significant covariates.

In the CON group, mean CTX-1 concentrations were 1.03 ± 0.6 ng/mL (95% CI: [0.62, 1.44]) at pre-intervention and 0.80 ± 0.4 ng/mL (95% CI: [0.51, 1.09]) at post-intervention, with no significant within-group difference (*p* = 0.05, *d* = 0.47, 95% CI: [−0.02, 0.96]). In the INT group, concentrations were 1.13 ± 0.5 ng/mL (95% CI: [0.79, 1.46]) at pre-intervention and 1.12 ± 0.5 ng/mL (95% CI: [0.77, 1.46]) at post-intervention, with no significant within-group difference (*p* = 0.84, *d* = 0.05, 95% CI: [−0.41, 0.51]).

There was no significant between-group differences in change scores (ΔCTX-1) (*t*(20) = 1.2, *p* = 0.24, *d* = 0.52, 95% CI: [−0.38, 1.42]). The CON group had a mean change of −0.23 ± 0.4 ng/mL (95% CI: [−50.1, 49.6]), while the INT group had a mean change of −0.01 ± 0.4 ng/mL (95% CI: [−60.5, 60.5]).

### 3.5 Cytokine and inflammatory markers

#### 3.5.1 sRANKL

**Figure 4:**
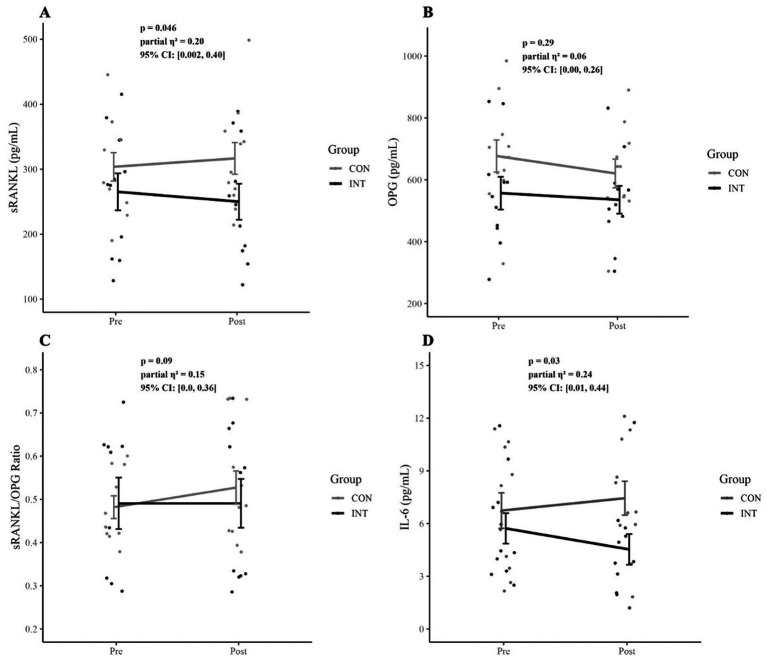
Changes in cytokine and inflammatory markers from pre- to post-intervention. **(A)** Soluble receptor activator of nuclear factor *κ*B ligand (sRANKL), **(B)** osteoprotegerin (OPG), **(C)** sRANKL/OPG ratio, and **(D)** interleukin-6 (IL-6) in the control (CON) and intervention (INT) groups. Data are presented as individual participant values, with group means + standard error. Statistical results for time group interactions are displayed within each panel.

**Figure 5:**
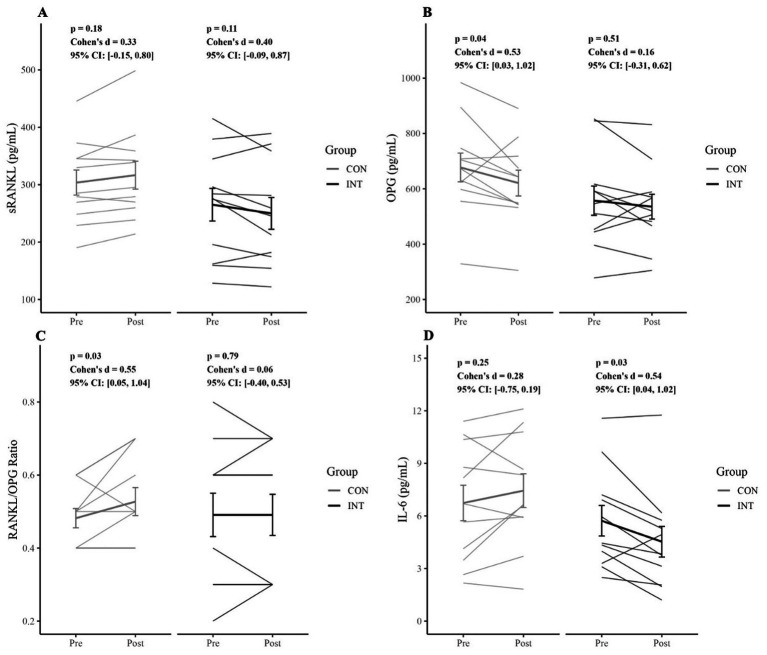
Within-group changes in cytokine and inflammatory markers from pre- to post-intervention. **(A)** Soluble receptor activator of nuclear factor *κ*B ligand (SRANKL), **(B)** osteoprotegerin (OPG), **(C)** SRANKL/OPG ratio, and **(D)** interleukin-6 (IL-6) in the control (CON) and intervention (INT) groups. Data are presented as individual participant trajectories with group means standard error. Within-group pre-post comparisons are shown for each condition.

Changes in serum sRANKL concentrations across the intervention period are shown in Table 6 and Figures 4, 5. There was a significant group × time interaction (*F*(1, 18) = 4.59, *p* = 0.046, *η*^2^_p_ = 0.20, 95% CI: [0.002, 0.40]. There were no significant main effects for group (*F*(1, 18) = 1.68, *p* = 0.21, *η*^2^_p_ = 0.09, 95% CI: [0.00, 0.29], or time (*F*(1, 18) = 1.34, *p* = 0.26, *η*^2^_p_ = 0.07, 95% CI: [0.00, 0.27]. Neither accumulated running distance (*p* = 0.27) nor baseline 25(OH)D_3_ status (*p* = 0.84) were significant covariates.

**Table 6:** Serum RANKL, OPG, RANKL/OPG ratio, and IL-6 concentrations at pre- and post-intervention.

Variable	CON (*n* = 11)		INT (*n* = 11)		*p*-values			Effect size *η*^2^_p_ [95% CI]
	Pre	Post	Pre	Post	Group	Time	Interaction	
sRANKL (pg/mL)	303 ± 73	316 ± 81	265 ± 94	250 ± 92	0.21	0.26	0.046	0.20 [0.002, 0.40]
OPG (pg/mL)	677 ± 172	620 ± 154	557 ± 175	535 ± 148	0.17	0.12	0.29	0.06 [0.00, 0.26]
sRANKL/OPG Ratio	0.48 ± 0.09	0.53 ± 0.13	0.49 ± 0.19	0.49 ± 0.18	0.83	0.44	0.09	0.15 [0.00, 0.36]
IL-6 (pg/mL)	6.74 ± 3.4	7.4 ± 3.2	5.72 ± 2.9	4.53 ± 2.9	0.11	0.51	0.03	0.23 [0.01, 0.43]

Values represent mean ± SD; *p*-values reflect results from separate repeated-measures ANCOVAs adjusted for accumulated running distance and baseline 25(OH)D₃ status. Effect sizes are presented as partial eta-squared (*η*^2^_p_) with 95% confidence intervals. RANKL: receptor activator of nuclear factor-κB ligand; OPG: osteoprotegerin; IL-6: interleukin-6.

Mean sRANKL concentrations in the CON group were 303 ± 73 pg/mL (95% CI: [255, 352]) at pre-intervention and 316 ± 81 pg/mL (95% CI: [262, 371] at post-intervention, with no significant within-group difference (*p* = 0.17, *d* = 0.33, 95% CI: [−0.15, 0.80]). In the INT group, mean values were 265 ± 94 pg/mL (95% CI: [202, 328]) at pre- and 250 ± 92 pg/mL (95% CI: [188, 312]) at post-intervention, with no significant within-group difference (*p* = 0.11, *d* = 0.40, 95% CI: [−0.09, 0.87]).

Change scores (ΔsRANKL) revealed a significant between-group difference (*t*(20) = 2.6, *p* = 0.02, *d* = 1.1, 95% CI: [0.15, 2.1]), with mean changes of 12.9 ± 20.1 pg/mL (95% CI: [−0.6, 26.5]) in CON and −15.1 ± 29.6 pg/mL (95% CI: [−35.0, 4.7]) in INT (Table 7). When controlling for accumulated distance and baseline 25(OH)D_3_ status, these between-group differences remained significant (*F*(1, 18) = 6.63, *p* = 0.02, *η*^2^_p_ = 0.27, 95% CI: [0.02, 0.46].

**Table 7:** Change in RANKL and OPG concentrations, RANKL/OPG ratio, and IL-6 from pre- to post-intervention.

Variable	CON (*n* = 11)	INT (*n* = 11)	Between groups *p*-value	Effect size Cohen’s *d* [95% CI]
Δ sRANKL (pg/mL)	12.9 ± 20.1	−15.1 ± 29.6	0.02	1.1 [0.15, 2.06]
Within-group *p*-value	0.18	0.11		
Δ OPG (pg/mL)	−56.3 ± 96.3	−30.7 ± 85.4	0.37	0.39 [−0.50, 1.29]
Within-group *p*-value	0.04	0.51		
Δ sRANKL/OPG Ratio	0.05 ± 0.08	0.00 ± 0.06	0.16	0.62 [−0.29, 1.53]
Within-group *p*-value	0.03	0.79		
Δ IL-6 (pg/mL)	0.70 ± 1.6	−1.19 ± 1.4	0.01	1.25 [0.27, 2.2]
Within-group *p*-value	0.25	0.04		

Values represent mean ± SD. Between-group *p*-values determined using independent samples *t*-tests with equal variance assumed. Within-group *p*-values derived from RM-ANCOVA post hoc comparisons with Bonferroni adjustment; ΔsRANKL: change in (ng/mL); ΔOPG: change in (ng/mL); INT: intervention group; CON: control group; Cohen’s *d* and corresponding 95% confidence intervals reflect between-group effect sizes.

#### 3.5.2 OPG

Changes in serum OPG concentrations across the intervention period are shown in Table 6 and Figures 4, 5. There were no significant main effects for group (*F*(1, 18) = 2.07, *p* = 0.17, *η*^2^_p_ = 0.10, 95% CI: [0.00, 0.31], or time (*F*(1, 18) = 2.65, *p* = 0.12, *η*^2^_p_ = 0.13, 95% CI: [0.00, 0.33], and no significant group × time interaction (*F*(1, 18) = 1.21, *p* = 0.29, *η*^2^_p_ = 0.06, 95% CI: [0.0, 0.26]). Neither accumulated running distance (*p* = 0.64) nor baseline 25(OH)D_3_ status (*p* = 0.92) were significant covariates.

In the CON group, mean OPG concentrations decreased from 677 ± 172 pg/mL (95% CI: [562, 793]) at pre-intervention to 620 ± 154 pg/mL (95% CI: [517, 724] at post-intervention, representing a significant within-group difference (*p* = 0.04, *d* = 0.53, 95% CI: [0.03, 1.02]). In the INT group, mean values were 557 ± 175 pg/mL (95% CI: [439, 675]) at pre- and 535 ± 148 pg/mL (95% CI: [435, 635]) at post-intervention, with no significant within-group difference (*p* = 0.51, *d* = 0.16, 95% CI: [−0.31, 0.62]).

Change scores (ΔOPG) revealed no significant between-group difference (*t*(20) = 0.92, *p* = 0.37, *d* = 0.39, 95% CI: [−0.50, 1.29]), with mean change values of −56.3 ± 96.3 pg/mL (95% CI: [−121.0, 8.5]) in CON and −30.7 ± 85.4 pg/mL (95% CI: [−74.7, 31.4]) in INT.

#### 3.5.3 sRANKL/OPG ratio

Changes in sRANKL/OPG across the intervention period are shown in Table 6 and Figures 4, 5. A repeated-measures ANCOVA revealed no significant main effects for group (*F*(1, 18) = 0.05, *p* = 0.83, *η*^2^_p_ = 0.003, 95% CI: [0.00, 0.10], or time (*F*(1, 18) = 0.62, *p* = 0.44, *η*^2^_p_ = 0.03, 95% CI: [0.00, 0.21], and no significant group × time interaction (*F*(1, 18) = 3.25, *p* = 0.09, *η*^2^_p_ = 0.15, 95% CI: [0.0, 0.36]. Neither accumulated running distance (*p* = 0.29) nor baseline 25(OH)D_3_ status (*p* = 0.58) were significant covariates.

In the CON group, mean sRANKL/OPG values increased from 0.48 ± 0.09 AU (95% CI: [0.42, 0.54]) at pre-intervention to 0.53 ± 0.13 AU (95% CI: [0.44, 0.61] at post-intervention, representing a significant within-group difference (*p* = 0.03, *d* = 0.55, 95% CI: [0.05, 1.04]). However, in the INT group, mean values were 0.49 ± 0.19 AU (95% CI: [0.36, 0.62]) at pre-intervention and 0.49 ± 0.18 AU (95% CI: [0.35, 0.62]) at post-intervention, with no significant within-group difference (*p* = 0.79, *d* = 0.06, 95% CI: [−0.40, 0.53]).

Change scores (ΔsRANKL/OPG) revealed no significant between-group difference (*t*(20) = 1.46, *p* = 0.16, *d* = 0.62, 95% CI: [−0.29, 1.53]), with mean change values of 0.05 ± 0.08 AU (95% CI: [−0.01, 0.10]) in CON and 0.00 ± 0.06 AU (95% CI: [−0.04, 0.04]) in INT (Table 7).

#### 3.5.4 IL-6

Changes in serum IL-6 concentrations across the intervention period are shown in Table 6 and Figures 4, 5. There was a significant group × time interaction (*F*(1, 18) = 5.77, *p* = 0.03, *η*^2^_p_ = 0.24, 95% CI: [0.01, 0.44]. There were no significant main effects for group (*F*(1, 18) = 2.83, *p* = 0.11, *η*^2^_p_ = 0.14, 95% CI: [0.00, 0.34] or time (*F*(1, 18) = 0.46, *p* = 0.51, *η*^2^_p_ = 0.03, 95% CI: [0.00, 0.19]. Neither accumulated running distance (*p* = 0.42) nor baseline 25(OH)D_3_ status (*p* = 0.26) were significant covariates.

In the CON group, mean IL-6 concentrations were 6.74 ± 3.4 ng/mL (95% CI: [4.48, 8.99]) at pre-intervention and 7.4 ± 3.2 ng/mL (95% CI: [5.29, 9.59] at post-intervention, with no significant within-group difference (*p* = 0.25, *d* = 0.28, 95% CI: [−0.75, 0.19]). In the INT group, mean values decreased from 5.72 ± 2.9 ng/mL (95% CI: [3.79, 7.66]) at pre-intervention to 4.53 ± 2.9 ng/mL (95% CI: [5.29, 9.59]) at post-intervention, representing a significant within-group difference (*p* = 0.03, *d* = 0.54, 95% CI: [0.04, 1.02]).

Change scores (ΔIL-6) revealed a significant between-group difference (*t*(20) = 2.93, *p* = 0.01, *d* = 1.25, 95% CI: [0.28, 2.22]), with mean changes of 0.70 ± 1.6 ng/mL (95% CI: [−0.01, 0.10]) in CON and −1.19 ± 1.4 ng/mL (95% CI: [−2.11, −0.28]) in INT (Table 7). When controlling for accumulated distance and baseline 25(OH)D_3_ status with a one-way ANCOVA, these between group differences remained significant (*F*(1, 18) = 19.84, *p* = 0.01, *η*^2^_p_ = 0.34, 95% CI: [0.06, 0.51].

No adverse events or unintended effects related to the intervention were reported during the study.

## 4 Discussion

This exploratory randomized, double-blind, placebo-controlled pilot trial examined the effects of 4 weeks of CP supplementation on bone turnover, cytokine, and inflammatory markers in premenopausal female runners. This study is novel in that it represents the first short-term intervention to investigate CP supplementation on bone turnover dynamics within a young, endurance-trained female cohort, an underrepresented population in clinical bone research and applied sport nutrition literature. The primary findings indicate that CP supplementation increased circulating P1NP, suggesting enhanced type I collagen synthesis and bone formation, and reduced IL-6 concentrations, consistent with attenuation of inflammatory signaling. While CTX-1 did not demonstrate parallel reductions, stabilization of the sRANKL/OPG ratio in the intervention group, suggests a potential early modulation of osteoclastogenic signaling. These data provide preliminary evidence that short-term CP supplementation may favorably influence bone formation and osteoimmune regulation in endurance-trained premenopausal women.

### 4.1 PINP

For P1NP, a significant group × time interaction and large effect size was observed, indicating that changes in bone formation activity differed between groups. This interaction was driven by a within-group increase in P1NP in the INT group, whereas no change was observed in the CON group. Previous investigations into the effects of CP supplementation on P1NP have yielded inconsistent results across populations, dosages, and intervention durations. To date, only one other study has examined this relationship in a mixed-sex endurance athlete cohort that included premenopausal women (50). In that 18-week open-label trial, elite cyclists (age range: 16–35 years) consumed 15 g of CPs followed by a brief (~5 min) plyometric exercise program five times per week. Participants were randomized to CP or a no-treatment CON group. In contrast to the present findings, P1NP concentrations declined by ~17% in both groups from pre- to post-intervention, yielding a significant main effect of time, but no time × treatment interaction (50). Notably, the sample included both women (*n* = 28) and men (*n* = 8), and analyses were not stratified by sex, limiting interpretation of sex-specific responses in P1NP (50). Additionally, differences in habitual mechanical loading may have influenced outcomes. Cycling imposes minimal osteogenic strain (51), and although the intervention included a plyometric protocol involving multidirectional hopping and vertical jumping (50), it is unclear whether the duration, frequency, or cumulative load of this exercise program was sufficient to support a bone-forming responses in conjunction with CP supplementation. In contrast, distance running imposes repetitive, weight-bearing impacts with high ground-reaction forces (52), which may better prime the skeleton for an osteoanabolic effect of CP supplementation (50).

In postmenopausal women with osteopenia, prior studies have demonstrated divergent effects of CP supplementation on P1NP, with both reductions and increases reported depending on study context. Argyrou et al. (62) observed a 13% decrease in P1NP following 3 months of CP supplementation (5 g/day) combined with calcium and vitamin D, while Lampropoulou-Adamidou et al. (63) reported a similar 17% reduction after 12 months under comparable co-supplementation conditions (51, 52). In contrast, König et al. (2018) found that 12 months of CP supplementation (5 g/day) without calcium or vitamin D significantly increased P1NP by 11.6% relative to placebo (18). Taken together, these findings indicate that CP supplementation does not elicit a uniform effect on bone formation markers, but rather that the direction of responses may depend on baseline remodeling status and the presence of co-interventions. The increase in P1NP observed in the present study aligns directionally with the findings of König et al., despite differences in population (postmenopausal vs. premenopausal), intervention duration (12 months vs. 4 weeks), and training status. In contrast, the reductions reported by Argyrou et al. (62) and Lampropoulou-Adamidou et al. (63) differ from the present findings and may reflect the influence of calcium and vitamin D co-supplementation, which are known to suppress bone turnover in postmenopausal populations (53–55). Additionally, baseline P1NP concentrations in those studies were substantially higher than those observed in the current cohort, suggesting a physiological context characterized by elevated turnover, where reductions in P1NP may reflect normalization rather than suppression of bone formation. In the present study, which involved endurance-trained premenopausal women not receiving co-supplementation, the observed increase in P1NP may instead reflect a context-specific anabolic response to CPs under conditions of habitual mechanical loading and comparatively lower baseline turnover. However, the absence of studies directly comparing CP supplementation with and without calcium and vitamin D in premenopausal populations limits definitive conclusions and represents an important area for future investigation.

### 4.2 CTX-1

For CTX-1, no significant group × time interaction or between-group differences were observed, indicating that CP supplementation did not meaningfully alter bone resorption activity in the present study. This finding is consistent with the broader literature, in which CP supplementation has generally demonstrated minimal or no effect on CTX-1 across a range of populations, intervention durations, and dosing strategies (51, 56, 57).

Evidence from both observational and interventional studies supports this pattern. In a mixed-sex cohort that included female runners, (56) reported no changes in post-exercise CTX-1 concentrations following 16 weeks of CP supplementation (56). Similarly, short-term exercise-based trials in young men have consistently shown no effect of CP supplementation on CTX-1. Clifford et al. (20) observed no changes following 9 days of supplementation combined with a bout of high-impact plyometric exercise, while (50) reported no changes after 3 days of high-impact jumping with CP intake (20, 48). Notably, these studies also failed to demonstrate changes in P1NP, suggesting a broader absence of remodeling response under short-term conditions in these populations. Collectively, these findings indicate that CP supplementation alone, particularly over short durations, may exert limited influence on bone resorption activity. In this context, the absence of change in CTX-1 in the present study aligns with the prevailing pattern of null findings and supports the interpretation that the observed increase in P1NP may reflect a formation-driven response rather than a coupled remodeling effect.

It is also important to consider that CTX-1 is highly sensitive to acute physiological factors independent of supplementation. For example, (53) demonstrated that ingestion of glucose, fat, protein, or fructose resulted in rapid reductions in serum CTX of approximately 39–52% from baseline, an effect mediated in part by gut-derived peptides such as GLP-2. This suppression occurred independent of macronutrient composition, indicating that feeding itself exerts a potent inhibitory effect on bone resorption (53). In addition, CTX-1 exhibits marked circadian variation and is responsive to recent exercise exposure (58, 59). Although the present study controlled for fasting status and recent exercise prior to blood sampling, residual variability related to habitual dietary intake or training patterns may have attenuated the ability to detect subtle changes in CTX-1.

Finally, while the present findings align with studies demonstrating minimal effects of CP supplementation on CTX-1, evidence from exercise-based models suggests a more nuanced interaction between collagen intake and mechanical loading. In resistance-trained and middle-aged men, ingestion of hydrolyzed collagen in conjunction with resistance exercise has been shown to augment P1NP in a dose-dependent manner, while CTX decreases transiently following exercise independent of collagen dose (54, 55). These findings indicate that collagen supplementation may preferentially enhance collagen synthesis pathways without directly modifying resorption, particularly in the acute post-exercise period. Together, this body of work suggests that the effects of CP supplementation on bone turnover may be asymmetric, favoring formation-related processes, and may depend on the interaction between nutritional intake and mechanical stimuli rather than supplementation alone.

### 4.3 Cytokine and inflammatory markers

Regarding changes in cytokine activity across the 4-week intervention, a significant group × time interaction was observed for sRANKL, alongside a significant between-group difference in ΔsRANKL, indicating a divergent response between conditions. Specifically, sRANKL concentrations decreased in the INT group and increased in the CON group, suggesting a potential attenuation of osteoclastogenic signaling with CP supplementation. In contrast, no interaction effects were observed for OPG or the sRANKL/OPG ratio. However, OPG decreased significantly within the CON group while remaining unchanged in the INT group, a pattern that may reflect a shift toward a more pro-resorptive environment in the absence of supplementation. Taken together, these findings suggest that CP supplementation may influence osteoclast-related signaling, although the lack of consistent effects across all markers warrants cautious interpretation.

To date, no human studies have directly examined the effects of CP supplementation on sRANKL or OPG. Preclinical models provide relevant mechanistic context, demonstrating that CPs can increase OPG expression while suppressing sRANKL signaling in osteoblasts, thereby shifting the balance toward reduced osteoclastogenesis (9, 60, 61). This effect has been observed across multiple concentrations and time points *in vitro*, supporting a direct influence of CP-derived peptides on osteoblast-mediated regulation of bone resorption. While translation to *in vivo* human systems remains uncertain, the reduction in sRANKL observed in the INT group, alongside the stability of OPG compared to its decline in the CON group, is directionally consistent with these mechanistic findings.

For IL-6, a significant group × time interaction was observed, with concentrations decreasing in the INT group and remaining unchanged in the CON group. This finding contrasts with much of the existing literature, which has generally reported null or context-dependent effects of CP supplementation on inflammatory markers. For example, (19) observed no change in IL-6 following 12 weeks of supplementation in older adults, although IL-6 increased in the placebo group over time, suggesting a potential attenuation of low-grade inflammation. In exercise-based models, (20) reported no significant effects of CP supplementation on IL-6 following acute high-intensity exercise, although magnitude-based inference suggested a possible early-phase attenuation of the post-exercise response. Similarly, (21) found no evidence of CP-mediated modulation of IL-6 following prolonged treadmill exercise, with both groups exhibiting comparable increases from baseline.

In this context, the reduction in IL-6 observed in the present study suggests a potential anti-inflammatory effect of CP supplementation under conditions of sustained endurance training, which may differ from the acute or lower-load conditions examined in prior work (19–21). However, given the inherent variability of cytokine responses and the limited number of sampling timepoints, this finding should be interpreted cautiously. While CP supplementation may influence inflammatory signaling, the consistency and underlying mechanisms of this response remain to be established.

### 4.4 Additional considerations

An additional consideration when interpreting these findings is the potential influence of endogenous sex hormones. In premenopausal women, fluctuations in estrogen across the menstrual cycle are known to influence both bone turnover and inflammatory markers, with lower estrogen levels generally associated with increased bone resorption and alterations in cytokine activity (22). In the present study, testing sessions were standardized within the early follicular phase (i.e., within the first 7 days of the menstrual cycle), a period characterized by relatively low and stable estrogen concentrations, to minimize variability associated with cyclical hormonal fluctuations. However, circulating hormone concentrations were not directly measured due to resource constraints. As such, while cycle phase was controlled, interindividual differences in hormone levels within this phase may have contributed to variability in biomarker responses.

### 4.5 Limitations

Several limitations should be considered when interpreting findings from the current study. First, P1NP and CTX-1 are surrogate biomarkers and do not directly reflect structural or densitometric adaptations. In addition, the four-week intervention period was insufficient to capture downstream changes in bone mass or microarchitecture. Second, although key preanalytical variables were standardized, including a requirement to refrain from exercise in the 24 h prior to sampling, bone turnover and cytokine markers are inherently variable and may still be influenced by habitual training patterns and interindividual differences in training status. Third, inflammatory assessment was limited to a narrow cytokine panel and two sampling timepoints, restricting insight into broader immunological dynamics and temporal responses. Finally, the study was not prospectively registered. However, all primary and secondary outcomes were defined *a priori*.

## 5 Conclusion

In summary, this pilot trial provides preliminary evidence that 4 weeks of high-dose CP supplementation may favorably modulate bone formation and inflammatory signaling in endurance-trained premenopausal women. CP intake increased P1NP without altering CTX-1, suggesting a formation-driven response, and was accompanied by stabilization of the sRANKL/OPG ratio and a reduction in circulating IL-6. Together, these findings support the biological plausibility that CPs may influence osteoblast activity and osteoimmune regulation in a population exposed to repetitive mechanical loading and elevated skeletal stress. Although limited by sample size, short duration, and absence of imaging-based outcomes, the magnitude and direction of the observed effects justify further investigation. Larger, longer-term trials incorporating structural bone measures and expanded mechanistic biomarkers are warranted to determine whether these early biochemical changes translate into meaningful skeletal adaptations and reduced injury risk in female endurance athletes.

## Data Availability

The raw data supporting the conclusions of this article will be made available by the authors, without undue reservation.
